# Factors affecting intention to breastfeed among Syrian and Jordanian mothers: a comparative cross-sectional study

**DOI:** 10.1186/1746-4358-5-6

**Published:** 2010-07-02

**Authors:** Nemeh Ahmad Al-Akour, Mohammad Yousef Khassawneh, Yusuf S Khader, Alla Ahmad Ababneh, Azeiza M Haddad

**Affiliations:** 1 Department of Maternal-Child Health Nursing, School of Nursing, Jordan University of Science and Technology (JUST), Irbid 22110, Jordan; 2 Pediatric Department, Faculty of Medicine, Jordan University of Science and Technology (JUST), Irbid 22110, Jordan; 3 Department of Community Medicine, Public Health and Family Medicine, Faculty of Medicine, Jordan University of Science and Technology (JUST), Irbid 22110, Jordan; 4 School of Nursing, Jordan University of Science and Technology (JUST), Irbid 22110, Jordan

## Abstract

**Background:**

Breastfeeding is considered the ideal method of infant feeding for at least the first six months of life. This study aimed to compare breastfeeding intention between Syrian and Jordanian women and determine factors associated with breastfeeding intention among pregnant women in these two countries.

**Methods:**

A cross-sectional design was used to collect data from1200 pregnant women aged 18 years and above (600 participants from each country). A self- administered questionnaire was used to collect data on socio-demographic characteristics and breastfeeding intention.

**Results:**

Intention to breastfeed was reported by 77.2% of Syrian and 76.2% of Jordanian pregnant women. There was no significant difference in intention to breastfeed between Syrian women and Jordanian women. In both countries, women with a more positive attitude to breastfeeding, women with previous breastfeeding experience and women with supportive partners were more likely to intend to breastfeed. Syrian women with a monthly family income of more than US$200, younger than 25 and primiparous or having one child were more likely to report an intention to breastfeed their infants. Jordanian women with an education level of less than high school and not living with their family-in-law were more likely to intend to breastfeed.

**Conclusions:**

In Syria and Jordan, a more positive attitude to breastfeeding, previous breastfeeding experience and presence of supportive husbands are associated with intention to breastfeed. These factors should be considered when planning programs designed to promote breastfeeding in these two countries.

## Background

Breastfeeding is considered the ideal method of feeding and nurturing infants [1]. Infants who are not breastfed have increased rates of mortality and increased risk of several chronic childhood diseases [2]. One and a half million deaths among infants could be avoided each year if all infants were breastfed exclusively during the first six months of life [3]. Breastfeeding intention is a significant predictor of infant feeding method; Tarrant *et al *recently reported that breastfeeding intention is a significant predictor of breastfeeding initiation and any breastfeeding at six weeks among Irish women [4]. Studies have described many factors associated with the intention to breastfeed [5-10]. These factors include maternal age, mother's education level, family household income, number of children, mother's knowledge about the benefits of breastfeeding, previous breastfeeding experience, attitude towards breastfeeding and the mother's social support network. Understanding factors associated with intention to breastfeed will allow health care decision-makers to plan and evaluate appropriate interventions to improve breastfeeding initiation and duration.

In the current study, we examined the intention to breastfeed among pregnant women in Jordan and Syria. These two neighboring Arab countries are similar in language and religion and share many socio-demographic characteristics. Infant feeding data from UNICEF demonstrated minor differences in breastfeeding practices between these two countries [11,12]. Although early initiation (39% and 32% in Jordan and Syria), and rates of exclusive breastfeeding to six months (22% and 29%, respectively) are similarly low in both countries, the proportion of children receiving breastfeeding and complementary food at 6-9 months was higher in Jordan (66%) than in Syria (37%). The 2007 Jordan population and family health survey (JPFHS), showed that only 40% of mothers exclusively breastfed their infants during the first five months of life, and about 11% were never breastfed [13]. The health systems in these two countries are similar in many aspects, as most of the general population in both Jordan and Syria receive health care through governmental agencies. Health education on breastfeeding mainly comes in the two countries through UNICEF programs including the Baby Friendly Hospital Initiative. This study aimed to compare breastfeeding intention between Syrian and Jordanian women and determine factors associated with intention to breastfeed among pregnant women in these two countries.

## Methods

### Study population

This study was conducted between 1 May 2008 and 1 October 2008. A sample of 1200 Syrian and Jordanian pregnant women aged 18 years and above, who had a normal pregnancy as determined by obstetricians, was included in the current study. Participants were able to speak and read Arabic. Of the 12 main health centers in Lattakia City, Syria, six centers were randomly selected. Six hundred Syrian women were then recruited from these centers. These centers are representative of the main maternal and child health centers in the northwest of Syria and serve populations with different levels of socio-economic status. Using simple random sampling techniques, six maternal and child health (MCH) centers out of 40 MCH centers in Irbid directorates in Jordan were selected. A sample of 600 Jordanian pregnant women was selected using the "proportionate to size" method. A total of 1200 participants from all pregnant women attending targeted health centers in both countries were recruited during the study period. Six trained nurses were responsible for identifying mothers who met the eligibility criteria for participating in the study. Informed consent was obtained from all participants after providing adequate information about the purposes of the study.

### Data collection

A self-administered questionnaire was used to collect data in both countries. The questionnaire consisted of three sections. The first section included data on participants' socio-demographic characteristics and related breastfeeding characteristics including: maternal age (years), level of education, monthly family income, number of live births, previous breastfeeding experience, breastfeeding education, occupation, whether living with a partner or not, maternal smoking during pregnancy, household composition, and breastfeeding support. The second section included one question with five items (plans) derived from a questionnaire developed by Humphreys and colleagues to assess breastfeeding intention [14]: 1) I am going to bottle-feed my baby, and I do not want to breastfeed at all. 2) I am thinking about breastfeeding, but I am not sure I want to do it. 3) I plan to try breastfeeding, but I am not sure how long I will do it. 4) I plan to breastfeed my baby for at least 1 month but probably not a full 6 months. 5) I plan to breastfeed my baby for at least 6 months. Women were defined as not having the intention to breastfeed if they reported "I am going to bottle-feed my baby, and I do not want to breastfeed at all" or "I am thinking about breastfeeding, but I am not sure I want to do it". Women were defined as having the intention to breastfeed if they reported any of the items 3, 4, 5. The third section assessed women's infant feeding attitudes using the Iowa Infant Feeding Attitude Scale (IIFAS) [15]. The IIFAS has been demonstrated to be both reliable and valid [9,15] and consists of 17 items that are scored on a 5-point Likert scale. The scale ranges from 1 (strongly disagree) to 5 (strongly agree). Eight items reflect positive attitudes to breastfeeding and nine items reflect positive attitudes to formula feeding. Items that favored formula feeding were scored reversely and a total attitude score was computed via an equally weighted sum of responses to all items. Total attitude scores range from 17 to 85, with higher scores indicating positive attitudes to breastfeeding [15]. Cronbach's alpha was 0.75 and 0.70 among Syrian and Jordanian participants respectively.

### Instrument translation

Translation of the instruments from the English version into the Arabic version was conducted to ensure that content, semantic, and technical equivalence were retained. Back translation of the instruments was conducted to ensure the original (English) and the second language (Arabic) version of the instruments measured the same words and concepts [16,17].

### Data analysis

Data were analyzed using the Statistical Package for Social Sciences (SPSS) windows version 15. Chi-square was used to compare the proportion of women who intended to breastfeed according to the socio-demographic and relevant characteristics. Multivariate binary logistic regression analyses were performed to determine the variables related to the intention to breastfeed among participants in the two groups. A p-value of 0.05 or less was considered as statistically significant.

## Results

### Participants' characteristics

One thousand two hundred Jordanian and Syrian pregnant women (600 women from each country) participated in this study. The age of participants ranged from 18 to 45 years. The mean age (SD) of participants were 27.9 (6.5) and 28.7 (5.6) years for Syrian and Jordanian women respectively. The mean (SD) IIFAS score of participants were 3.6 (0.4) and 3.3 (0.3) for Syrian and Jordanian women respectively. The characteristics of the participants are shown in Table 1.

**Table 1:** Socio-demographic and relevant characteristics of the Syrian and Jordanian pregnant women

Variables	Syria		Jordan	
	n	%	n	%

Mothers age (years)				
≤ 25	254	42.2	186	31.0
> 25	346	57.7	414	69.0
Education				
< high school	406	67.7	51	8.5
≥high school	194	32.3	549	91.5
Occupation				
Employed	104	17.3	110	18.3
Unemployed	496	82.7	490	81.7
Smoking				
Yes	185	31.8	67	11.2
No	415	69.2	533	88.8
Family income per month				
≤ US$ 200	335	55.8	382	63.7
> US$ 200	265	44.2	218	36.3
Household composition				
Not living with family in law	519	86.5	539	89.8
Living with family in law	81	13.5	61	10.2
Number of live birth				
0 (Primiparous)	151	25.2	192	32
1	165	27.5	100	16.7
2	160	26.7	113	18.8
≥3	124	20.7	195	32.5
Previous breastfeeding experience				
Yes	410	68.3	354	59.0
No	190	31.7	246	41.0
Breastfeeding husband support				
Yes	565	94.2	481	80.2
No	35	5.8	119	19.8
Breastfeeding family/peer support				
Yes	492	82	424	70.7
No	108	18	176	29.3
Breastfeeding intention				
No	137	22.8	143	23.8
Yes	463	77.2	457	76.2

### Intention to breastfeed

Intention to breastfeed was reported by 77.2% of Syrian participants and 76.2% of Jordanian participants. There was no significant difference in the odds of breastfeeding intention between Syrian women and Jordanian women in the univariate analysis (OR: 1.06; 95% CI: 0.81, 1.38; p-value = 0.68) and in the multivariate analysis (OR: 0.91; 95% CI: 0.62, 1.34; p-value = 0.64) after adjusting for relevant factors including: maternal age, level of education, income, previous breastfeeding experience, number of live births, and attitude to breastfeeding. Intention to breastfeed in both countries in relation to different socio-demographic and relevant variables is shown in Table 2. Breastfeeding intention among Syrian women was more common among women aged > 25 years, women with an education level of high school or more, employed women, women with monthly income of > US$ 200, women with < 3 live births, and women with previous breastfeeding experience. Among Jordanian women, breastfeeding intention was more common among those with education of less than high school, non-smokers, women not living with their family-in-law, women having live births of one or more, women who heard about breastfeeding from health professionals, women with previous breastfeeding experience, and women who had breastfeeding support from their husbands.

**Table 2:** Breastfeeding intention according to socio-demographic characteristics among Syrian and Jordanian mothers

Variables	Syria			Jordan		
	
	Do not intend to breastfeed n (%)	Intend to breastfeed n (%)	P-value	Do not intend to breastfeed n (%)	Intend to breastfeed n (%)	P-value
Mothers age(years)			0.08			0.12
≤ 25	67 (26.4)	187 (73.6)		87 (26.3)	244 (73.7)	
> 25	70 (20.2)	276 (79.8)		56 (20.8)	213 (79.2)	
Education			0.01			0.01
< high school	106 (26.1)	300 (73.9)		5 (9.8)	46 (90.2)	
≥ high school	31 (16)	163 (84)		138 (25.1)	411 (74.9)	
Occupation			0.05			0.21
Employed	16 (15.4)	88 (84.6)		22 (20.0)	88 (80.0)	
Not employed	121 (24.4)	375 (75.6)		121 (24.7)	369 (75.3)	
Smoking			0.96			0.00
Yes	42 (22.7)	143 (77.3)		30 (44.8)	37 (55.2)	
No	95 (22.9)	320 (77.1)		113 (21.2	420 (78.8)	
Family income per month			0.01			0.32
≤ $200	90 (26.9)	245 (73.1)		86 (22.5)	296 (77.5)	
> $200	47 (17.7)	218 (82.3)		57(26.1)	161 (73.9)	
Household composition			0.47			0.00
Not living with family in law	121 (23.3)	398(76.7)		117 (21.7)	422 (78.3)	
Living with family in law	16 (19.8)	65(80.2)		26 (42.6)	35 (57.4)	
Number of live births			0.01			0.00
0	41 (27.2)	110 (72.8)		63 (32.8)	129 (67.2)	
1	24 (14.5)	141 (85.5)		25 (25)	75 (75)	
2	34 (21.3)	126 (78.8)		13 (11.5)	100 (88.5)	
≥3	38 (30.6)	86 (69.4)		42 (21.5)	153 (78.5)	
Hearing about breastfeeding benefits			0.14			0.03
Media	8 (5.8)	53 (11.4)		88 (27.6)	231 (72.4)	
Relatives	81 (59.1)	246 (53.1)		39 (22.3)	136 (77.7)	
Health care professionals	48 (35.0)	164 (35.4)		16 (15.1)	90 (84.9)	
Previous breastfeeding experience			0.02			0.00
Yes	82 (20)	328 (80)		57 (16.1)	297 (83.9)	
No	55 (28.9)	135 (71.1)		86 (35)	160 (65)	
Breastfeeding partner support			0.01			0.00
Yes	125 (22.1)	440(77.9)		100 (20.8)	381 (79.2)	
No	12 (34.3)	23(65.7)		43 (36.1)	76 (63.9)	
Breastfeeding family/peer support			0.24			0.14
Yes	117 (23.8)	375 (76.2)		94 (22.2)	330 (77.8)	
No	20 (18.5)	88(81.5)		49 (27.8)	127 (72.2)	

### Factors associated with intention to breastfeed

The multivariate analysis of factors associated with breastfeeding intention in Syria and Jordan are shown in Table 3 and Table 4, respectively. In both countries, women with a positive attitude to breastfeeding, women with previous breastfeeding experience and women with supportive partners were more likely to intend to breastfeed. Syrian women with a monthly family income of more than US$200, younger than 25 years and primiparous or having one child were more likely to report an intention to breastfeed their infants. Jordanian women with an education level of less than high school and not living with their family-in-law were more likely to intend to breastfeed.

**Table 3:** Multivariate analysis of factors associated with breastfeeding intention among Syrian mothers

Variables	Adjusted Odds Ratio (95% Confidence Interval)	P-value
Mothers age(years)		
≤ 25	1.7 (1.03, 2.67)	0.04
> 25	1	
Family income per month		
≤ $200	1	
> $200	1.8 (1.15, 2.68)	0.01
Number of live births		
0	3.7 (1.41, 9.52)	0.01
1	3.9 (2.01, 7.36)	0.01
≥2	1	
Previous breastfeeding experience		
Yes	2.2 (1.04, 4.62)	0.04
No	1	
Breastfeeding partner support		
Yes	2.3 (1.06, 5.11)	0.04
No	1	
Positive attitude scores*	1.1 (1.049, 1.126)	0.01

* Total attitude scores range from 17 to 85, high scores indicated towards to positive breastfeeding attitude

**Table 4:** Multivariate analysis of factors associated with breastfeeding intention among Jordanian mothers

Variables	Adjusted Odds Ratio (95% Confidence Interval)	P-value
Education		
< high school	2.9 (1.17, 7.11)	0.01
≥ high school	1	
Previous breastfeeding experience		
Yes	1.6 (1.32, 2.11)	0.01
No	1	
Living with partner		
Yes	1.8 (1.11, 2.84)	0.02
No	1	
Household composition		
Not living with family in law	2.5 (1.55, 4.63)	0.01
Living with family in law	1	
Breastfeeding partner support		
Yes	2. 2 (1.47, 3.33)	0.01
No	1	
Positive attitude scores*	1.2 (1.15, 1.30)	0.01

* Total attitude scores range from 17 to 85, high scores indicated towards to positive breastfeeding attitude

## Discussion

The current study showed that there was no significant difference in the breastfeeding intention between Syrian women and Jordanian women. The majority of Syrian (77.2%) and Jordanian (76.2%) pregnant women intended to breastfeed their infants. This is similar to findings reported in studies in the United States of America (USA) [14,18]. Hill *et al. *found that 78% of respondents intended to breastfeed [18]. Humphreys *et al. *found that 53% of respondents intended to breastfeed [14]. In Greece, Ladomenou *et al. *[19] found that 89.7% of 1049 mothers intended to breastfeed. However, a study from Australia evaluated intention to exclusively breastfeed for six months and found that only 42% of women have this intention [20].

In this study, pregnant women were more likely to report an intention to breastfeed if they had positive attitudes toward breastfeeding. Our study results are consistent with the findings of other authors in USA [10] and United Kingdom [9] who found that breastfeeding intention is associated with positive breastfeeding attitudes. The findings of the current study showed that Syrian and Jordanian pregnant women were more likely to intend to breastfeed if they had previous breastfeeding experience. Humphreys *et al. *reported that previous breastfeeding experience was the strongest predictive factor associated with breastfeeding intention [5].

The results of this study suggested that Syrian and Jordanian pregnant women were more likely to intend to breastfeed if they had support of their husbands. This finding is in agreement with other studies that found that women who identified their husbands as the most important person who supported their breastfeeding decision were more likely to report intention to breastfeed [5,10,21,22]. This finding emphasizes the importance of including husbands in any breastfeeding promotion programs. The Qur'an, the Holy Book of Muslims, emphasizes the importance of breastfeeding frequently. In Islamic instruction, mothers are entitled a monthly payment from their husbands to breastfeed their children [23]. We can speculate that mothers' intention to breastfeed can be achieved by building a social environment that is supportive of breastfeeding.

Our finding indicated that Syrian women aged 25 or less were more likely to report an intention to breastfeed their infants than women older than 25 years. This finding may be explained by a new trend for more breastfeeding in the younger generation. This can also be an effect of breastfeeding promotion through media, inclusion of breastfeeding benefits in school curriculum or higher education in young Syrian women. In contrast, other studies have identified older maternal age as being strongly associated with breastfeeding intention [5-8]. However, age was not significantly associated with breastfeeding intention among Jordanian women. The present study showed that higher education in Jordan was associated with less breastfeeding intention. Our finding is in agreement with report of another study carried out in Jordan [24]. Khassawneh *et al. *found that mothers who had lower education were more likely to breastfeed than mothers with higher education [24]. In contrast, other studies from China and the United States of America [7,14] found that higher education was significantly associated with breastfeeding intention. This difference between Jordan and other countries can be explained by knowing that women with higher education in our culture are more likely to be working mothers and their intention to use bottle feeding is higher in a culture that provides minimal support for breastfeeding in the work environment. However, education was not significantly associated with breastfeeding intention among Syrian women. Unlike Western studies [25,26], our study showed that maternal smoking was not associated with breastfeeding intention.

Although socio-economic and demographic characteristics are similar among Syrian and Jordanian populations, the differences in intention between Syrian and Jordanian mothers could be explained by other factors. The provisional figures for the 2008 census give population figures of 5 million persons living in Jordan, and 13.8 million living in Syria, growing at rate of 2.47 percent per year in Jordan, and 3.21 per year in Syria. According to the 2003 estimate, the literacy rate was 91.3% of the total population in Jordan, while in Syria the 2003 estimate was 79.8% [27,28].

This study provided information about breastfeeding intention among Syrian and Jordanian mothers. The findings of this study emphasize the importance of partners' support for positive breastfeeding intention. Health policy makers in Syria and in Jordan can use findings in this study to increase rates of breastfeeding in these two populations. However, this study did not test whether the intention to breastfeed translates into breastfeeding practice. The results of this study invite further longitudinal studies in this aspect. Qualitative studies using focus groups can be helpful in providing more insight about barriers to breastfeed intention among pregnant women.

## Conclusions

Syrian women and Jordanian women were similar in their intention to breastfeed. In these two countries, breastfeeding intention was significantly associated with positive attitudes to breastfeeding, previous breastfeeding experience and the presence of supportive husbands. These factors should be considered when planning programs designed to promote breastfeeding in these two countries.

## Competing interests

The authors declare that they have no competing interests.

## Authors' contributions

NAA: Design, analysis, writing. MK: Design, analysis, writing. YSK: Design, analysis, writing methods and results. AAA: Design, data acquisition, writing manuscript. AMH: Design, data acquisition, writing manuscript. All authors have approved the final version of this manuscript.
